# Effects of glutamine and valsartan on the brain natriuretic peptide and N-terminal pro-B-type natriuretic peptide of patients with chronic heart failure

**DOI:** 10.12669/pjms.311.6302

**Published:** 2015

**Authors:** Mingliang Shao, Congxin Huang, Zhen Li, Hui Yang, Qifan Feng

**Affiliations:** 1 Dr. Mingliang Shao, Department of Cardiology, Renmin Hospital of Wuhan University, Cardiovascular Research Institute of Wuhan University, Wuhan, People’s Republic of China.; 2 Dr. Congxin Huang, Department of Cardiology, Renmin Hospital of Wuhan University, Cardiovascular Research Institute of Wuhan University, Wuhan, People’s Republic of China.; 3 Dr. Zhen Li, Department of Cardiology, Renmin Hospital of Xuancheng City, Xuancheng, People’s Republic of China.; 4 Dr. Hui Yang, Department of Cardiology, Renmin Hospital of Xuancheng City, Xuancheng, People’s Republic of China.; 5 Dr. Qifan Feng, Department of Cardiology, Renmin Hospital of Xuancheng City, Xuancheng, People’s Republic of China.

**Keywords:** Glutamine, Valsartan, Chronic heart failure, Brain natriuretic peptide, N-terminal pro-B-type natriuretic peptide

## Abstract

**Objective:**

To analyze the effects of glutamine and valsartan on the brain natriuretic peptide (BNP) and N-terminal pro-B-type natriuretic peptide (NT-proBNP) of patients with chronic heart failure (CHF).

**Methods:**

A total of 140 CHF patients were divided into a treatment group and a control group by random drawing, and were subjected to standard anti-heart failure treatment and administered with valsartan. Besides, the treatment group was also intravenously transfused glutamine. The treatment lasted eight weeks.

**Results:**

The overall efficacy of treatment group and control group were 98.6% and 90.0% respectively, with a statistically significant difference (P<0.05). The two groups had significantly increased left ventricular ejection fractions as well as significantly decreased left ventricular end-diastolic volumes and left ventricular end-diastolic dimensions after treatments (P<0.05) compared with those before. There were also inter-group differences between these values (P<0.05). After treatment, the levels of BNP, NT-proBNP and CD8^+^ in both groups significantly decreased (P<0.05), whereas those of CD4^+^ significantly increased (P<0.05). The two groups also had significantly different values (P<0.05).

**Conclusion:**

Glutamine in combination with valsartan enhanced the therapeutic effects by improving cardiac function, which may be associated with decreased expressions of BNP and NT-proBNP and beneficial effects of glutamine on immune function.

## INTRODUCTION

Chronic heart failure (CHF), which is a clinically common and complex syndrome, has poor prognosis for being the ending of a variety of organic heart diseases.^1^^,^^2^ Currently, CHF has become one of the severe diseases endangering human health with population aging and has thus attracted global attention. As a natriuretic endogenous peptide neurohormone, brain natriuretic peptide (BNP) is mainly secreted by ventricular myocytes, the synthesis of which is closely related with ventricular afterload and wall tension.^3^^,^^4^ Besides, N-terminal pro-B-type natriuretic peptide (NT-proBNP) contains 108 amino acids after truncating 26 of them at the N-terminal.^5^^,^^6^

In human body, BNP is active and decomposes rapidly, but the inactive NT-proBNP remains stable and thus allows easy detection.^7^ It is now well-established that over-activations of the renin-angiotensin-aldosterone system (RAAS) and the sympathetic nervous system dominantly control the vicious circle of cardiac remodeling and the deterioration of heart function.^8^ Accordingly, it is of great significance to inhibit the over-activation and vicious circle of neurohumor, and to reverse ventricular remodeling.^9^ Up to now, angiotensin II receptor blockers (ARBs) are drugs that specifically block Ang II receptors, effectively inhibit Ang II or aldosterone escape, and provide clinical benefits for heart failure patients. Of all ARBs, valsartan has been widely used to treat heart failure.^10^

On the other hand, glutamine, a main immune nutrient, provides nitrogen source for the synthesis of amino acids, proteins and nucleic acids. Upon severe wasting diseases such as trauma, heart failure, major surgery and cancer, human body begins to require a large amount of glutamine that, if not supplemented in time, will give rise to various diseases when exhausted.^11^^,^^12^ Therefore, we herein analyzed the influences of glutamine and valsartan on the BNP and NT-proBNP of CHF patients.

## METHODS

***Subjects: ***A total of 140 CHF patients enrolled in our hospital from September 2010 to January 2014 were selected in this study.

***Inclusion criteria:*** The patients conforming to the diagnostic standards for CHF; the patients suffering from history or clinical manifestations of congestive heart failure for three months; the patients who had New York Heart Association (NYHA) Class III-IV; the patients with written consent.

***Exclusion criteria:*** The patients with hepatic and renal insufficiency or myocardial infarction within three months; the patients who had received or were planning to receive heart transplantation; the patients who had stroke within three months; the patients having thyroid disease, blood disorders, endocrine disorders, or connective tissue disease. There were 77 males and 63 females who were aged 41-79 years old (average: 66.44 ± 13.73). NYHA classification: 80 cases of Class II, 30 cases of Class III, and 30 cases of Class IV; Disease type: 76 cases of coronary artery disease, 34 cases of dilated cardiomyopathy, 18 cases of hypertensive heart disease, and 12 cases of rheumatic heart disease. They were then divided into a treatment group and a control group by random drawing (n=70), and their basic data such as gender, age, NYHA classification and disease type were similar (P>0.05).

***Treatment methods:***

***Control group:*** This group was subjected to standard anti-heart failure treatment with ARBs, Digitalis and diuretics (capoten, hydrochlorothiazide and digoxin: 25 mg each, qd), and was administered valsartan (Jiangsu Kanion Pharmaceutical Co., Ltd., National Medicine Permit No. H20090262, 80 mg, qd) simultaneously for eight consecutive weeks.

***Treatment group:*** In addition to the drugs mentioned above, this group was also intravenously administered glutamine (Sino-Swed Pharmaceutical Co., Ltd., National Medicine Permit No. H20053409, 0.5 mg/kg) by a micro-infusion syringe pump at the speed of 4-5 ml/h for eight consecutive weeks, once a week.

***Observation indices: ***Detection of BNP and NT-proBNP: Blood (3 ml) was intravenously collected, added into an EP tube containing EDTA-Na_2_, and centrifuged at 3000 rpm for 10 min to separate the plasma. The levels of BNP and NT-proBNP were detected by the enzyme linked immunoassay with a quick bedside testing instrument (Bosch, USA).

***Detection of cardiac function:*** Left ventricular end-systolic dimension (LVESD), left ventricular end-diastolic dimension (LVEDD) and left ventricular ejection fraction (LVEF) were detected before and after treatment by an experienced sonographer with a Vivid 7 color Doppler ultrasonography (GE, USA).

***Evaluation on therapeutic effects:*** Markedly effective: Basically controlled clinical symptoms and recovery of over two NYHA classes; effective: gradually controlled clinical symptoms and recovery of one NYHA class; ineffective: uncontrolled clinical symptoms and recovery of less than one NYHA class or even deterioration.

***Detection of T-lymphocyte subsets:*** Plasma BNP was collected and measured with a kit purchased from Biosino Bio-Technology & Science Inc. (China), with the levels of CD4^+^ and CD8^+^ as the main indices.

***Statistical analysis: ***The data were analyzed by SPSS 18.0. The numerical data were expressed as (mean ± standard deviation) and compared by independent samples t-test and paired t test, and the categorical data were expressed as absolute number or percentage and compared with the Chi-square test. P<0.05 was considered statistically significant.

## RESULTS

***Changes in levels of BNP and NT-proBNP: ***After treatment, the levels of BNP and NT-proBNP decreased significantly compared with those before (P<0.05), and the treatment group had significantly lower values than the control group did (P<0.05) (Table-I).

***Changes in cardiac function indices: ***The two groups had significantly increased LVEFs as well as significantly decreased LVEDVs and LVEDDs after treatments (P<0.05) compared with those before. Moreover, there were also inter-group differences between these values (P<0.05) (Table-II).

***Overall therapeutic effects: ***The overall effective rates of the treatment group and the control group were 98.6% and 90.0% respectively, with a statistically significant difference (P<0.05) (Table-III).

***Changes in T-lymphocyte subsets: ***After treatment, the levels of CD8^+^ in both groups significantly decreased (P<0.05), whereas those of CD4^+^ significantly increased (P<0.05). Meanwhile, the two groups also had significantly different values (P<0.05) (Table-IV).

## DISCUSSION

CHF, as the end stage of heart diseases originating from various reasons other than a simple hemodynamic disorder, involves many neurohormonal factors that keep worsening this disease.^13^ Traditionally; CHF is treated targeting hemodynamic disorders, which mitigates symptoms by being heart-strengthening, diuretic and blood vessel-dilating. However, this protocol fails to stop the development of CHF or to raise the survival rate.^14^ The onset and development of heart failure stem from ventricular remodeling on which the activation of neuroendocrine exerts adverse regulating effects. Particularly, long-term over-activation of RAAS predominantly participates in cardiac remodeling and progressive deterioration of CHF.^15^ As a specific Ang II receptor-blocking agent; valsartan can postpone the development of cardiovascular disease by effectively inhibiting Ang II or aldosterone escape, with the maximum effective rate of only about 90% though.^16^

On the other hand, glutamine is crucial for the metabolism and synthesis of nucleic acids and mainly energizes the metabolic activities of intestinal mucosal cells, so supplementing exogenous glutamine has evident protective effects on the intestinal mucosal barrier.^17^ In the meantime, it is able to stimulate human body to produce glucagon and to boost the intestinal immune function. In this study, the overall effective rates of the treatment group and the control group were 98.6% and 90.0% respectively, with a statistically significant difference (P<0.05). It has been reported that glutamine can enhance anti-oxidative capacity, promote intestinal tract movement, improve state of nutrition, and thus benefit the recovery of cardiac function.^18^ Besides, glutamine maintains the integrity of intestinal mucosa, protects the structure and function of intestinal mucosa, prevents endotoxin from entering the blood circulation, and decreases the expressions of inflammatory cytokines and endotoxin, thus reducing the risk of multi-system organ damage.

The onset and development of CHF involve activation of the sympathetic nervous system and enhanced synthesis and release of BNP from the dilated atrium and ventricle. When over-activated, BNP can dilate the blood vessels and facilitate the natriuresis of CHF patients by regulating the specific receptors of the kidney and vascular smooth muscle.^19^ Upon severe heart failure, however, this beneficial effect is compensated by vasoactive substance-induced powerful vasoconstriction and sodium retention, thereby leading to poor diagnosis.

It is now well-known that CHF results in ventricular remodeling by elevating cardiac load. As a result, more NT-proBNP is released because of increased wall tension. Actually, NT-proBNP is not released from necrotic cardiac muscle. Instead, it mainly originates from the survival cardiac muscle under augmented local tension. Additionally, increase in the degree of coronary artery stenosis, which is associated with the increase in involved blood vessels, is able to alarm the degree of CHF.^20^ In this case, abundant glutamine supplies intestinal mucosal cells with necessary energy and raw materials, reduces the formation of cell membrane lipid peroxides, and thus minimizes the damage to cardiac function. In general, it contributes to the release of some hormones, elevates the activity of intestinal glutaminase, stimulates the pancreas and choleresis, resists the oxidative injury of oxygen free radicals to biomembrane, and increases the survival rate of cells. The two groups had significantly increased LVEFs as well as significantly decreased LVEDVs and LVEDDs after treatments (P<0.05) compared with those before. In addition, there were also inter-group differences between these values (P<0.05).

CHF patients are prone to body function decline and immune afunction that may induce vicious circle by leading to intestinal hypoperfusion and translocation of normal flora into blood. Under this circumstance, glutamine supplementation improves gastrointestinal functions by enhancing immune function and by boosting nutritional status. Furthermore, supplementation of exogenous glutamine can alleviate vigorous decomposition and metabolism, circumvent its deficiency under stress, facilitate a variety of synthesis and metabolism, and exert immune-enhancing effects.^15^ In this study, the levels of CD8^+^ in both groups significantly decreased after treatment (P<0.05), whereas those of CD4^+^ significantly increased (P<0.05). The two groups also had significantly different values (P<0.05).

**Table-I:** Changes in levels of BNP and NT-proBNP (x ± s).

**Group**	**Case number (n)**	**BNP (ng/L)**		**NT-proBNP (fmol/ml)**	
		**Before**	**After**	**Before**	**After**
Treatment group	70	298.98±20.83	45.93±12.17	4.56±0.78	2.61±0.45
Control group	70	300.32±21.62	98.37±13.87	4.59±0.67	3.44±0.67
t		0.287	7.984	0.387	9.331
P		<0.05	<0.05	<0.05	<0.05

**Table-II:** Changes in cardiac function indices (x ± s

**Group**	**Case number (n)**	**LVEF (%)**		**LVEDV (mm)**		**LVEDD (mm)**	
		**Before**	**After**	**Before**	**After**	**Before**	**After**
Treatment group	70	35.56±8.24	45.87±4.91	249.64±45.95	165.09±32.98	93.65±15.96	61.67±16.33
Control group	70	35.56±8.47	40.98±5.33	252.52±65.87	206.87±40.83	93.78±16.09	74.96±16.00
t		0.000	4.983	0.329	7.948	0.034	9.223
P		>0.05	<0.05	>0.05	<0.05	>0.05	<0.05

**Table-III:** Overall therapeutic effects (n).

**Group**	**Case number (n)**	**Markedly effective**	**Effective**	**Ineffective**	**Overall effective rate**
Treatment group	70	60	9	1	98.6%
Control group	70	40	23	7	90.0%
χ²					5.442
P					<0.05

**Table-IV:** Changes in T-lymphocyte subsets (%, x ± s).

**Group**	**Case number (n)**	**CD4** ^+^		**CD8** ^+^	
		**Before**	**After**	**Before**	**After**
Treatment group	70	35.93±2.98	50.98±3.18	31.37±4.71	19.76±4.78
Control group	70	36.00±3.67	45.39±3.76	31.78±4.26	25.96±4.10
t		0.287	5.898	0.438	7.001
P		<0.05	<0.05	<0.05	<0.05

In summary, valsartan, when combined with glutamine, has satisfactory therapeutic effects on CHF patients by being conducive to cardiac function, which is related with the decreased expressions of BNP and NT-proBNP as well as the beneficial effects of glutamine on immune function.

## Authors Contribution:

**MLS & CXH **conceived, designed and did statistical analysis & editing of manuscript.

**ZL** **,** **HY** **&** **QFF** did data collection and manuscript writing.

**CXH** did review and final approval of manuscript.

**CXH** takes the responsibility and is accountable for all aspects of the work in ensuring that questions related to the accuracy or integrity of any part of the work are appropriately investigated and resolved.
